# Supplementary material for: Associations between the red blood cell distribution width-to-albumin ratio and 3-month outcomes in patients with acute minor ischemic stroke: A cohort study
Source: PLoS One. 2025 Jul 28;20(7):e0329211. doi: 10.1371/journal.pone.0329211 (PMC12303272; doi:10.1371/journal.pone.0329211)
Supplement: Attachment — Submitted filename: Response to Reviewers - PONE-D-25-24758.docx [file pone.0329211.s003.docx]

Dear Dr. Ufuk Demirci and Reviewers,

We sincerely appreciate your thoughtful feedback on our manuscript entitled "Associations between the red blood cell distribution width-to-albumin ratio and 3-month outcomes in patients with acute minor ischemic stroke: A cohort study". The insights provided by you and the reviewers have been invaluable in guiding us to improve the manuscript. We have carefully considered each suggestion and strived to implement comprehensive revisions, hoping these adjustments align with the journal’s standards and your expectations. Revised sections are highlighted with red track changes in the manuscript. Below is a summary of the key modifications and our responses to the reviewers’ comments:

**Journal requirements:**

When submitting your revision, we need you to address these additional requirements.

1. Please ensure that your manuscript meets PLOS ONE's style requirements, including those for file naming. The PLOS ONE style templates can be found at

https://journals.plos.org/plosone/s/file?id=wjVg/PLOSOne_formatting_sample_main_body.pdf and

https://journals.plos.org/plosone/s/file?id=ba62/PLOSOne_formatting_sample_title_authors_affiliations.pdf

**Response:** We sincerely appreciate your invaluable suggestions provided during the review process. These suggestions have been instrumental in enabling us to enhance the manuscript to a greater extent. Concerning the format requirements of the journal, we have carried out a thorough and meticulous check, and subsequently made all the necessary revisions to ensure full compliance.

2. PLOS requires an ORCID iD for the corresponding author in Editorial Manager on papers submitted after December 6th, 2016. Please ensure that you have an ORCID iD and that it is validated in Editorial Manager. To do this, go to ‘Update my Information’ (in the upper left-hand corner of the main menu), and click on the Fetch/Validate link next to the ORCID field. This will take you to the ORCID site and allow you to create a new iD or authenticate a pre-existing iD in Editorial Manager.

**Response:** We sincerely appreciate your patient reminder and detailed guidance! We have now completed the update and validation of the ORCID iD as required. If there are any oversights or areas for improvement in the subsequent process, please kindly let us know. We will fully cooperate to rectify and perfect them.

3. Your ethics statement should only appear in the Methods section of your manuscript. If your ethics statement is written in any section besides the Methods, please delete it from any other section.

**Response:** We have confirmed the ethics statement is exclusively retained in the Methods section and removed from all other sections as required.

4. Please review your reference list to ensure that it is complete and correct. If you have cited papers that have been retracted, please include the rationale for doing so in the manuscript text, or remove these references and replace them with relevant current references. Any changes to the reference list should be mentioned in the rebuttal letter that accompanies your revised manuscript. If you need to cite a retracted article, indicate the article’s retracted status in the References list and also include a citation and full reference for the retraction notice.

**Response:** Thank you for your guidance regarding the reference list. We have carefully and thoroughly reviewed each citation in the manuscript, striving to ensure its completeness and accuracy. To the best of our knowledge, no retracted papers were identified among the references cited.

**Reviewer #1:** The study looks at the association between red cell distribution width-to-albumin ratio and three month mortality among acute minor ischemic stroke patients.

The article is very well written and features sound statistical analysis. I would recommend the following minor corrections:

Line 172: Please consider elaborating "SVO" as small-vessel occlusion upon first mention in the text. While it is defined in the accompanying table, introducing the full term at first use in the main text would improve clarity.

**Response:** Thank you for your valuable suggestion. At Line 166, when making reference to 'SVO', we have already presented the complete term 'small-vessel occlusion'. This measure has been taken to improve the readability of the main text, ensuring that readers can easily understand the context without ambiguity.

Line 274: The term "hemidiaphragm injury" appears to be an error. Please review this reference, as it may be a misstatement or require clarification.

**Response:** Thank you for catching this error. We corrected "hemidiaphragm injury" to "ischemic penumbra" at Line 269, aligning with standard terminology. Much appreciated for your careful review!

**Reviewer #2:** This is a well-conducted and clearly presented cohort study examining the association between the red blood cell distribution width-to-albumin ratio (RA) and 3-month outcomes in patients with acute minor ischemic stroke. The methodology is appropriate, and the statistical analyses are sound. The findings contribute meaningfully to the growing literature on prognostic biomarkers in stroke.

I have no major concerns and recommend acceptance of the manuscript. However, it would strengthen the discussion if the authors could briefly elaborate on how their findings might be translated into clinical care or influence clinical decision-making. For instance, how might RA be used in risk stratification or management of patients with minor stroke?

**Response:** Thank you for the suggestion. We added a paragraph in the Discussion (Lines 278-283) to discuss the RA ratio’s clinical implications in risk stratification and minor stroke management. Appreciate your insight!
